# Transformers in surgical artificial intelligence: A domain-stratified, study-level narrative review

**DOI:** 10.1016/j.xjon.2026.101597

**Published:** 2026-01-22

**Authors:** Andres Bravo, Sara Razzaq, Mohan Murari, Aditya Ahuja, Danielle Birchett, Lana Schumacher

**Affiliations:** Division of Thoracic Surgery, Department of Surgery, Tufts University, Boston, Mass

**Keywords:** artificial intelligence, medical education, medical imaging, prognosis, surgery, surgical workflow, transformers

## Abstract

**Objective:**

The objective was to synthesize study-reported outcomes of transformer-based artificial intelligence systems in surgical domains and describe settings where they appear advantageous relative to nontransformer models and human benchmarks.

**Methods:**

We searched major databases: PubMed, Embase, IEEE Xplore, ScienceDirect, Google Scholar, arXiv, and Cochrane Library. Eligible studies evaluated transformer architectures in surgical/perioperative contexts (medical imaging, workflow recognition, prognosis-related modeling, or education) and reported quantitative outcomes. Because of heterogeneous tasks/metrics, we performed a domain-organized narrative synthesis. Where the same study reported transformers and nontransformers on the same dataset/metrics, we computed within-study deltas (Δ = Transformers – Nontransformers) and summarized medians and interquartile ranges alongside vote counts (T>NT/tie/T<NT). No cross-study pooling or hypothesis testing was performed.

**Results:**

Paired comparisons favored transformers in medical imaging for 15 of 20 (75.0%) with median Δ +1.13 percentage points (interquartile range, 3.70) and in workflow recognition for 28 of 34 (82.4%) with median Δ +1.75 percentage points (interquartile range, 3.28). Prognosis had sparse paired data (n = 1; Δ +3.0 percentage points; illustrative). Education favored transformers overall in 5 of 6 (83.3%) paired comparisons, driven by surgery time prediction; diagnostic education tasks were mixed. Reported advantages were task dependent and dataset specific; gains were typically single-digit percentage points in like-for-like settings.

**Conclusions:**

Transformers frequently match or exceed nontransformer baselines in surgical imaging and workflow tasks, with promising, yet heterogeneously reported, signals in prognosis and education. Translation to dependable clinical/educational impact will require standardized benchmarks, external/prospective validation, transparent comparator reporting (including human baselines), and deployment studies that address real-time operating room constraints and fairness across patient and learner groups.

## Graphical Abstract

Transformers in surgical artificial intelligence: Study-level, domain-stratified narrative review using paired within-study differences (Δ = Transformer – Nontransformer) on the same dataset/metric. Results show consistent single-digit gains for imaging (median +1.13 percentage points, 15/20 comparisons favored transformers) and workflow imaging (median Δ +1.75 percentage points, 28/34 favored), with sparse/mixed evidence in prognosis and education. Implications: prioritize standardized benchmarks, external/prospective validation with human comparators on the same cases, and operating room–ready deployment addressing latency, and fairness.

**Figure:**
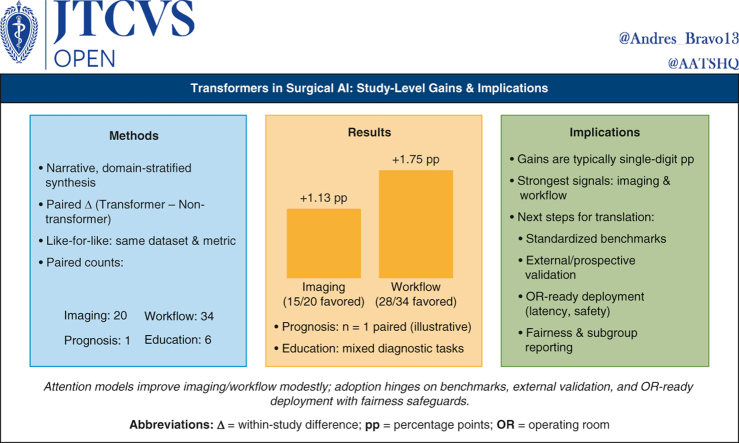

**Figure:**
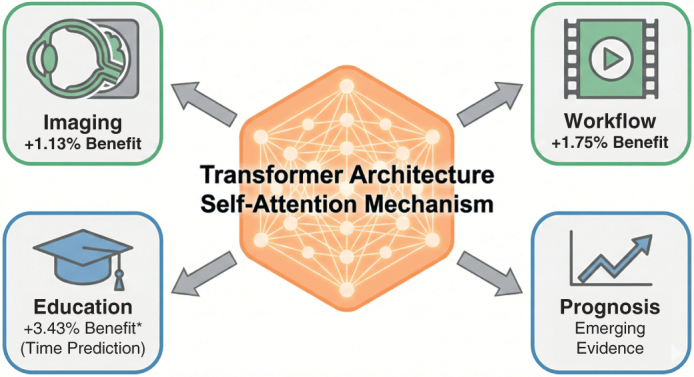
Self-attention yields consistent gains in surgical imaging and workflow tasks.

**Central Message**

Transformers outperform conventional models on surgical imaging/workflow in same-dataset tests; gains are modest but consistent. Impact hinges on benchmarks, external validation, and operating room deployment.

**Perspective**

Attention-based models capture long-range spatial/temporal context suited to imaging and workflow. Our study-level synthesis shows consistent single-digit gains over nontransformers. Translation demands standardized benchmarks, external/prospective validation with human comparators on the same cases, and evaluation of latency, safety, and fairness in operating rooms.

Artificial intelligence (AI) increasingly supports the surgical continuum, from preoperative planning to intraoperative guidance and postoperative risk prediction, while also enabling data-driven surgical education. Transformer architectures, centered on self-attention, have drawn particular interest for modeling long-range temporal/spatial dependencies and fusing heterogeneous perioperative data (images, video, text, and structured electronic health record [EHR] signals).^1^, ^2^, ^3^, ^4^, ^5^ Applications reported across the surgical literature include (1) medical image detection/segmentation/classification; (2) workflow understanding from operative video (phase/step/gesture recognition, tool presence, case duration prediction); (3) prognosis-related modeling (risk stratification, outcome prediction); and (4) educational assessment/feedback systems.^6^, ^7^, ^8^, ^9^, ^10^, ^11^, ^12^

Individual studies often report transformer performance that meets or surpasses conventional baselines, and selected reports compare favorably to human references. Yet others document negligible margins on simpler classification problems or note that nontransformer approaches remain competitive when data are limited, labels are coarse or imbalances, or latency/compute budgets dominate.^13^

Because task definitions, datasets, and metrics differ widely, pooled quantitative claims risk overgeneralization. Therefore, we adopt a domain-stratified narrative synthesis emphasizing that within-study, like-for-like comparisons on the same dataset/metrics differ widely, reporting descriptive medians/interquartile ranges (IQRs) of the Δ (Transformer – Nontransformer) and vote counts by domain. We do not perform cross-study pooling, re-estimation, or hypothesis testing. This approach foregrounds what each study demonstrates on its own terms and provides decision-relevant signals for surgeons, educators, and developers considering where transformers are most likely to help. The Graphical Abstract shows a visual summary of our approach and main findings.

## Background

### Scope and Task Taxonomy

We organize findings into 4 surgical/perioperative domains: (1) medical imaging (anatomy/tool/pathology detection, classification, segmentation); (2) workflow understanding (phase/step/gesture recognition, tool presence, case duration prediction) from video/sensors; (3) prognosis-related modeling (risk/outcome prediction from structured data and clinical text); and (4) medical education (AI-enabled assessment/feedback for knowledge and technical skills).

### Terminology and Comparators

“Transformer-based” denotes architectures whose primary representation mechanism relies on self-attention.^5^ “Nontransformer” comparators include convolutional neural networks, temporal convolutions/recurrent neural networks, and classic machine learning. “Human baselines” refer to expert/trainee performance as reported by each study. Metrics (eg, area under the curve [AUC], accuracy, precision/recall, harmonic mean of precision and recall [F1 score], and intersection over union [IoU]) are taken as reported.

### Measurement and Deployment Considerations

Interpretation depends on label granularity (frame vs segment vs case), class imbalance, dataset provenance/splits (internal vs external, temporal vs random), and real-time operating room constraints (latency budgets, occlusion, smoke/glare, device integration). Educational studies add learner heterogeneity and assessment fidelity. We reference these factors where relevant.

## Material and Methods

### Information Sources and Search Strategy

We queried PubMed, Embase, IEEE Xplore, ScienceDirect, Google Scholar, arXiv, and the Cochrane Library using predefined terms spanning transformer architectures and surgical/perioperative applications. We also screened reference lists of included articles.

### Eligibility Criteria

Included studies evaluated transformer-based models in surgical or perioperative domains and reported quantitative outcomes using standard metrics as defined by the original authors. When available, we recorded nontransformer and human comparators. We excluded nonevaluative pieces (eg, editorials) and studies without surgical/perioperative focus.

### Study Selection and Data Extraction

Titles and abstracts were screened for relevance; full texts were assessed against eligibility. From each included study, we extracted task/domain, model family/architecture, dataset characteristics, comparators (nontransformer or human), and study-reported evaluation metrics. Metrics are presented exactly as reported; no recalculation or re-estimation was performed.

### Synthesis Approach

Because of heterogeneity in tasks, label spaces, and outcome metrics, we conducted a narrative, domain-stratified synthesis. When a study reported both a transformer and a nontransformer on the same dataset and metric, we computed a within-study Δ = Transformer – Nontransformer and summarized medians and IQRs within the same domain, alongside vote counts (T>NT/tie/T<NT). For studies with multiple models per class, we used the best-performing model per class for that metric to form a single paired comparison; where multiple metrics were reported, deltas were computed per metric and summarized by metric within domain. Human baselines were treated as contextual comparators and not included in Δ calculations unless evaluated on the same cases. No cross-domain pooling, hypothesis testing, or re-estimation of study metrics was performed.

## Results

**Figure 1:**
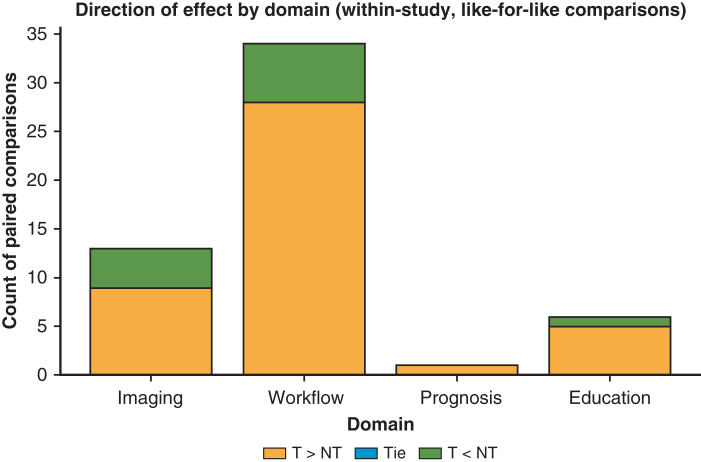
Direction of effect by domain (within-study, like-for-like comparisons). Stacked bars show the count of paired comparisons favoring transformers (T>NT), ties, or nontransformers (T<NT) within each domain. Comparisons are computed on the same dataset and outcome metric in each study. Descriptive only; not pooled effect sizes.

**Figure 2:**
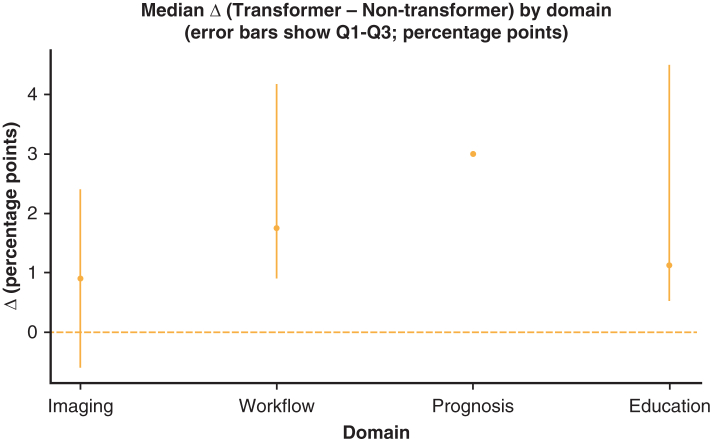
Median Δ (Transformer – Nontransformer) by domain (percentage points). Points show the median within-study Δ for each domain; error bars span the 25th to 75th percentiles (Q1-Q3). A *dashed line* at 0 indicates no difference. Descriptive only; not pooled effect sizes.

The overall direction of effect by domain is summarized in Figure 1, and domain-level medians of the within-study difference (Δ, percentage points) are shown in Figure 2.

### 1. Medical Imaging (Detection, Classification, Segmentation)

**Figure 3:**
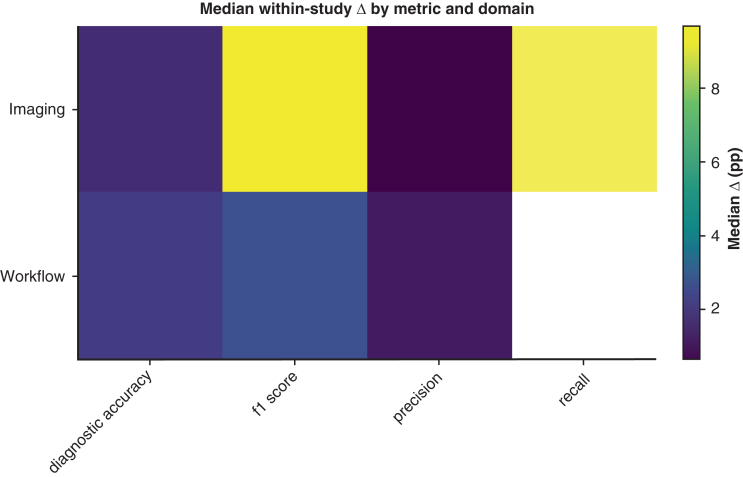
Median within-study Δ by metric and domain. Heatmap of median Δ (Transformer – Nontransformer, percentage points) for each metric (columns) within imaging and workflow (rows). This shows where advantages concentrate by metric family (eg, accuracy vs F1 score vs area under the receiver operating characteristic curve). Values are medians across paired comparisons; no cross-domain pooling or hypothesis testing.

**Figure E1:**
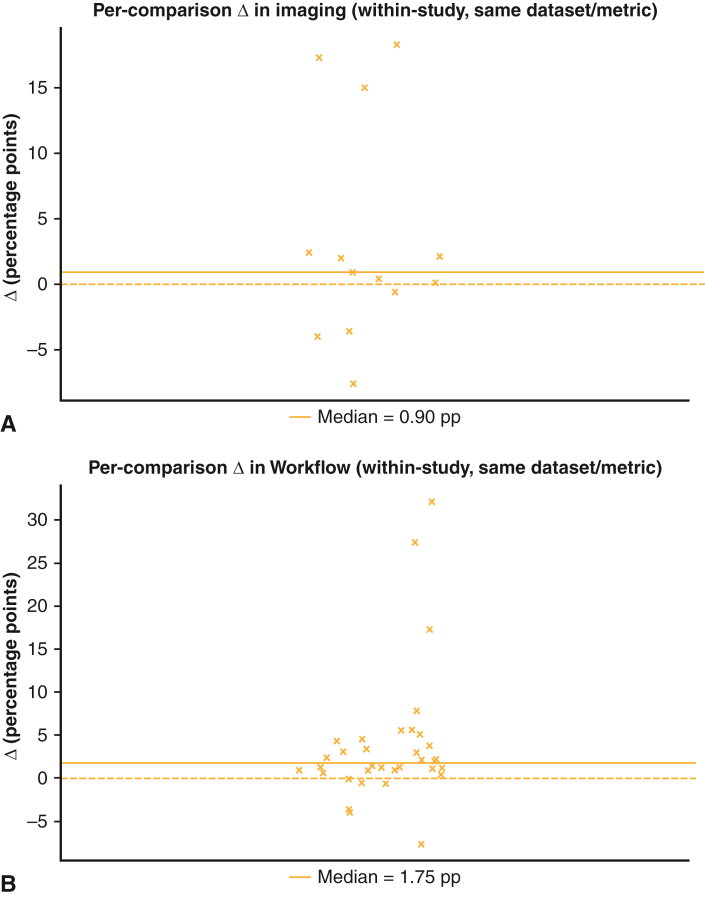
A, Per comparison Δ in imaging. Each *dot* is a within-study Δ (Transformer – Nontransformer) computed on the same dataset and outcome metric. *Solid line* shows the domain median; *dashed line* at 0 indicates no difference. B, Per comparison Δ in workflow. Each *dot* is a within-study Δ (Transformer – Nontransformer) computed on the same dataset and outcome metric. *Solid line* shows the domain median; *dashed line* at 0 indicates no difference.

**Figure E2:**
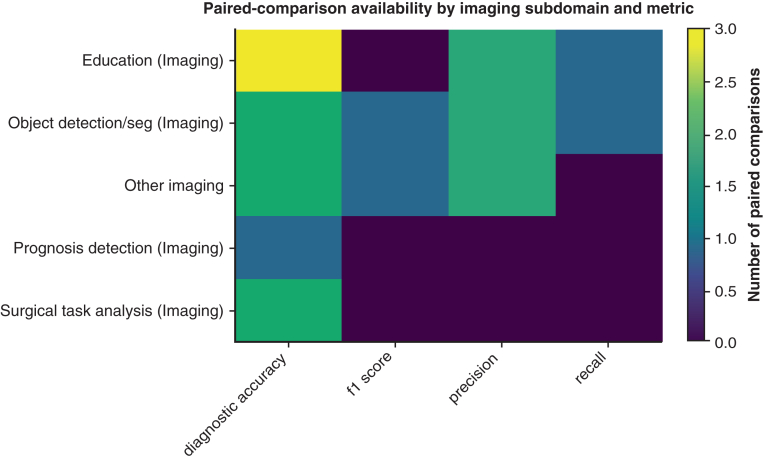
Paired-comparison availability by imaging subdomain and metric. Heatmap shows the count of within-study, like-for-like transformer–nontransformer comparisons in imaging, stratified by subdomain (rows) and metric (columns). *Darker cells* indicate more paired comparisons; blank/*lighter cells* indicate limited or no paired data. This visual highlights heterogeneity and data sparsely across metrics and tasks. Descriptive only.

Representative findings: On ATLAS stroke magnetic resonance imaging, a Shifted-Window Vision Transformer achieved 73.06% diagnostic accuracy, clearly exceeding SegNet (44.18%) and U-Net (48.31%) (≈+24.7-28.9 percentage points).^14^ On the lung image database consortium and image database resource initiative lung nodules, transformer models in Lai^15^ reported 82.15% accuracy (token-wise approximation) with 88.04% precision but modest F1 score (45.72%), reflecting class imbalance/label granularity; descriptive transformer averages across that study were 74.54% accuracy, 37% F1 score, and 82.94% precision. These task specifics illustrate where attention helps (context) and where dataset/labeling choices compress F1 score despite high precision.^15^ Metric-specific medians by domain are provided in Figure 3, which highlights where accuracy/F1 score/IoU gains are most consistent for imaging tasks. Figure E1, *A* shows within-study deltas computed on the same dataset and outcome metric and indicates the domain median compared with no difference. Additionally, we produced a heatmap (Figure E2) indicating the count of within-study, like-for-like transformer–nontransformer comparisons in imaging stratified by subdomain and metric to highlight heterogeneity and data sparsity. Detailed imaging counts by domain are provided in Table E1. Additional imaging counts organized by domain and metric are provided in Table E2.

Domain summary (paired like-for-like): A total of 15 of 20 (75.0%) paired comparisons favored transformers; median Δ +1.13 percentage points (IQR, 3.70) on the same dataset/metrics.

### 2. Workflow Understanding (Phase/Step/Gesture Recognition; Case Duration Prediction)

**Figure 4:**
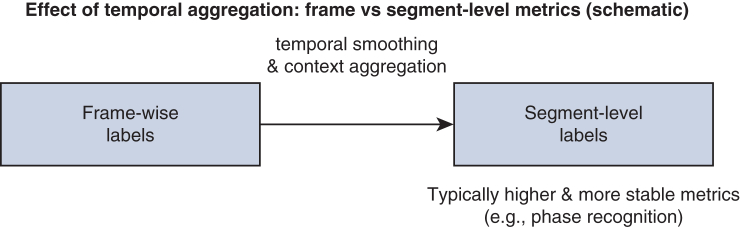
Effect of temporal aggregation: frame versus segment-level metrics (schematic). Conceptual illustration: segment-level labels (temporal smoothing and context aggregation) typically yield higher/stabler sequence-recognition metrics than frame-wise labels. No empirical data are plotted.

**Figure 5:**
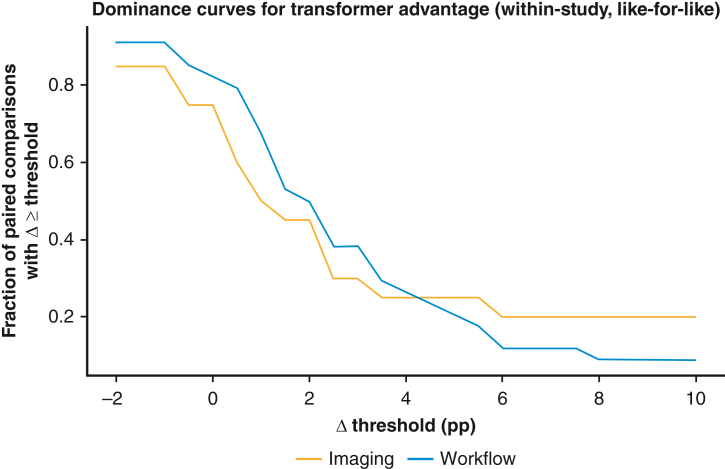
Operating room deployment considerations (schematic). Conceptual overview of real-time constraints that influence model reliability and translation: latency budgets, frame drops, tool occlusion, smoke/glare, throughput, and system integration.

For case duration prediction, clinical bidirectional encoder representations from transformers improved accuracy from 26.8% to 58.9% (+32.1 percentage points) versus historical nontransformer models.^16^ Detailed workflow counts by domain are reported in Table 1. Table E3 provides detailed workflow counts by domain and metric. In laparoscopic cholecystectomy phase recognition, the action segmentation temporal convolutional transformer formerly reported gains of +1.78% accuracy, +1.99% F1 score, and +2.24% precision versus nontransformers; with segmental (vs frame-wise) labels, it reached 95.67% accuracy and 92.31% precision (+6.84 and + 11.04 percentage points, respectively).^17^ For annotation, general surgery vision transformer achieved 86.3% accuracy/82.4% precision; transformer averages in that evaluation set were 85.56%/80.78%.^18^ For readers interested in why label granularity matters in video, Figure 4 contrasts frame- and segment-level labeling and how these choices surface transformer advantages. The distribution of advantages across thresholds (fraction of paired comparisons with Δ ≥ τ) is shown in Figure 5, illustrating that most observed gains are single-digit percentage points. Figure E1, *B* highlights within-study deltas computed on the same dataset and outcome metric and shows the domain median compared with no difference.

**Table 1:** Workflow (domain summaries)

Domain	T<NT	T>NT	Tie	N	Median Δ (percentage points)	IQR Δ (percentage points)
Education: Clinical documentation	0	3	0	3	5.1	12.95
Education: Medical diagnosis	1	0	0	1	−0.52	0.0
Education: Surgical automation	0	9	0	9	3.4	4.39
Object detection and segmentation	2	3	0	5	0.9	8.15
Prognosis detection	0	1	0	1	3.0	0.0
Surgical task analysis	2	3	0	5	1.24	1.42
Medical image analysis	1	9	0	10	2.05	2.25

*IQR,* Interquartile range.

**Figure 6:**
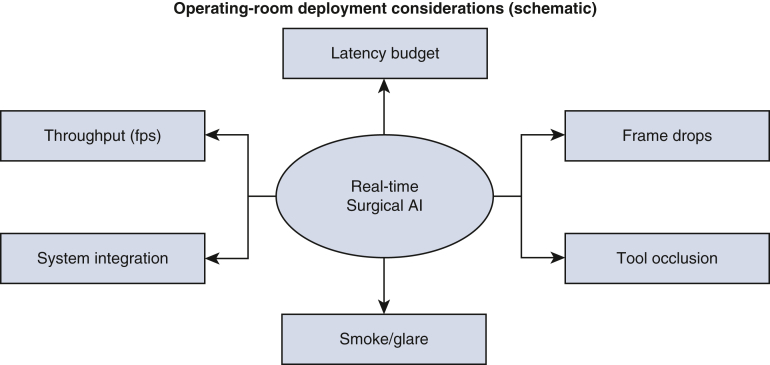
Dominance curves for transformer advantage (within-study, like-for-like). For each Δ threshold (x-axis, percentage points), the y-axis shows the fraction of paired comparisons with Δ ≥ threshold. Separate curves are shown for imaging and workflow. Curves above 0.5 indicate that a majority of studies meet or exceed that advantage at the specified threshold. Descriptive only; not a pooled effect.

Domain summary (paired like-for-like): 28/34 (82.4%) favored transformers; median Δ +1.75 percentage points (IQR, 3.28).

### 3. Prognosis-Related Modeling (Risk/Outcome Prediction; EHR/Natural Language Processing)

Evidence spans electronic medical record, notes, and multimodal signals. A nontransformer SERA algorithm reported 84% average accuracy versus 55.25% physicians for early sepsis with 21% to 32% earlier detection and 7% to 17% fewer false-positives (shows strong nontransformer baselines on some tasks).^19^ Natural language processing reviews note transformer area under the receiver operating characteristic curve up to 0.97 versus 0.83/0.81 for long short-term memory/support vector machine.^20^ Conversely, classic approaches can excel in narrow anomaly detection (eg, 98% on specific lung-abnormality tasks).^21^

Domain summary (paired like-for-like): n = 1 paired comparison (illustrative) favored transformers (Δ +3.0 percentage points); not generalizable.

### 4. Medical Education (Learner Outcomes; Assessment/Feedback)

AI-assisted testing improved multiple-choice scores by 32% over didactics in one study.^12^ Diagnostic education findings mirror underlying model behavior (eg, Lai's 2024 transformers^15^), but learner benefit also depends on curriculum/assessment fidelity.

Domain summary (paired like-for-like): 5 of 6 (83.3%) overall; driven by surgery-time prediction (4/4, median Δ +3.43 percentage points, IQR, 7.88). Diagnostic education tasks were mixed (½; median Δ −0.07 percentage points, IQR, 0.45).

## Discussion

### Synthesis of Findings

**Figure E3:**
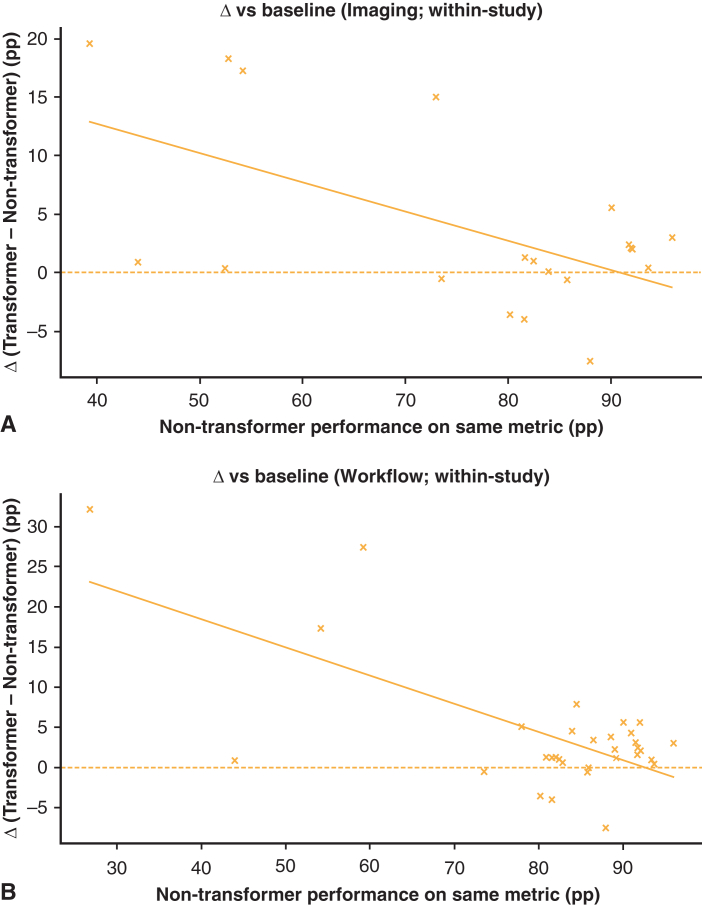
A, Relationship between *baseline* difficulty and Δ (within-study). Scatterplots show Δ (y-axis) versus the nontransformer baseline performance on the same dataset/metric (x-axis) for imaging (A) and workflow (B). A *fitted trend line* is displayed to visualize association (negative slope suggests diminishing gains as the baseline approaches ceiling performance). Each point is a paired comparison; descriptive only. B, Relationship between *baseline* difficulty and Δ (within-study). Scatterplots show Δ (y-axis) versus the nontransformer *baseline* performance on the same dataset/metric (x-axis) for imaging (A) and workflow (B). A *fitted trend line* is displayed to visualize association (negative slope suggests diminishing gains as the baseline approaches ceiling performance). Each point is a paired comparison; descriptive only.

Study-level evidence indicates task-dependent advantages for transformers, most consistently in workflow understanding (temporal context) and medical imaging requiring global spatial reasoning; signals are promising but heterogenous for prognosis and education. Gains are usually single-digit percentage points in paired like-for-like settings, although select tasks show double-digit jumps. Nontransformers remain competitive, and sometimes preferable, when (1) tasks are narrow or documentation oriented, (2) data are limited/imbalanced, or (3) latency/compute constraints dominate. Figure E3, *A* and *B* compares the delta versus nontransformer baseline performance on the same dataset/metric for imaging (A) and workflow (B).

### Where Transformers Help Most

(1) Long-range temporal modeling (phase/step/gesture), (2) multimodal fusion (images, video, text, structured EHR), (3) scaling to high-dimensional inputs, and (4) segment/episode-level labels that expose context (vs frame-wise). Reported improvements often expand when label granularity aligns with attention's global receptive field.

### Interpreting Comparators and Heterogeneity

“Human baselines” vary (expert vs trainee; same vs different subsets/times), so they are contextual rather than interchangeable controls. Evaluation protocols (internal vs external; random vs temporal splits) and metrics (accuracy, AUC, F1 score, IoU) differ widely. Our synthesis therefore limits claims to within-study, like-for-like comparisons, and domain-stratified descriptive summaries.

### Implications for Surgical Deployment

Few reports detail inference hardware, throughput, latency, or frame-drop behavior, critical for operating room integration. Robust translation will require (1) standardized benchmarks and transparent comparator reporting (including human baselines on the same cases); (2) external/prospective validation; (3) reporting calibration, error typologies, and subgroup/fairness metrics; and (4) evaluations that reflect real-time constraints, occlusion/smoke/glare, and integration with surgical platforms. Practical operating room considerations for deployment are summarized in Figure 6.

### Future Directions

Priority directions include multi-institutional, prospective evaluations; standardized benchmarks and reporting; robust generalization methods for dataset/case-mix shift; and deployment-focused studies that measure workflow impact, safety, and equity. For education, randomized or quasi-experimental designs with long-term retention outcomes will clarify true pedagogical value.

### Limitations

1. Heterogeneity of evidence. Tasks (eg, detection, segmentation, phase recognition, prognosis, education), datasets, labeling granularity (frame vs segment vs case), and evaluation protocols vary widely across studies. As a result, cross-study comparisons are descriptive only and should not be interpreted as pooled effects.

2. Reporting variability. Thresholds, class prevalence, and metric definitions (accuracy, AUC, precision/recall, F1 score, IoU, task-specific scores) are not standardized across articles. Many studies do not report calibration, confidence intervals, or error typologies, limiting interpretability and comparability.

3. Comparator inconsistency. “Human baselines” differ in expertise (experts vs trainees), setting, and sample size, and are not always evaluated on the same cases as the models. Nontransformer baselines likewise vary in quality and tuning. Consequently, comparator results are best treated as contextual rather than interchangeable controls.

4. Validation gaps. A substantial fraction of studies report internal validation only, sometimes with random (nontemporal) splits. External or prospective validation is inconsistent, which constrains conclusions about generalizability.

5. Real-world constraints underreported. Few studies provide sufficient detail on inference hardware, latency, throughput, frame-drop behavior, or integration considerations (eg, occlusion, smoke, glare) that are critical for operating room deployment.

6. Reproducibility and transparency. Code, trained weights, and detailed hyperparameters are inconsistently available; several reports rely on private datasets. These factors limit independent verification and re-use.

7. Bias and equity considerations. Demographic composition, case-mix, and site diversity are often underreported; fairness metrics and subgroup analyses are rarely provided. The degree to which results generalize across institutions and populations remains uncertain.

8. Risk of duplication within source literature. Multiple experiments from the same dataset or institution appear across different publications. Although this review does not pool results, readers should be cautious about implicit over-representation of particular cohorts.

9. Scope of synthesis. By design, we did not perform cross-study pooling, hypothesis testing, or re-estimation of effect sizes. This preserves fidelity to individual reports but precludes quantitative estimation of overall effect magnitude.

10. Search and selection constraints. Although multiple databases were queried, the search may not capture all relevant studies (eg, nonindexed venues, language restrictions, or very recent releases). We did not conduct a formal risk-of-bias or Grading of Recommendations Assessment, Development and Evaluation assessment, which would be valuable in future updates.

11. Paired-comparison availability and selection. Within-study delta analyses were possible only when studies reported both model classes on the same dataset/metric. We used each study's best-performing model per class for the paired comparison, which may modestly inflate differences relative to average performance.

12. Data extraction and harmonization. Although we cross-checked values against our study table, extraction and standardization across sources can introduce errors; any discrepancies identified postpublication will be corrected in an updated supplement.

## Conclusions

Transformer-based systems, as reported by individual studies, often outperform conventional approaches on complex surgical AI tasks, particularly those requiring temporal context and multimodal integration, whereas nontransformer methods remain competitive for narrower or resource-constrained problems. Given heterogeneity in tasks, datasets, and metrics, these findings should be interpreted as study-level evidence, not pooled estimates. Translating reported gains into dependable clinical and educational impact will require standardized benchmarks, external and prospective validation, transparent comparator reporting, and deployment studies that account for real-time operating room constraints and fairness across patient and learner groups.

## Conflict of Interest Statement

The authors reported no conflicts of interest.

The *Journal* policy requires editors and reviewers to disclose conflicts of interest and to decline handling or reviewing manuscripts for which they may have a conflict of interest. The editors and reviewers of this article have no conflicts of interest.

## Figures and Tables

**Table E1:** Imaging (domain summaries)

Domain	T<NT	T>NT	Tie	N	Median Δ (percentage points)	IQR Δ (percentage points)
Education: Medical diagnosis	1	1	0	2	−0.07	0.45
Education: Surgery time prediction	0	4	0	4	3.43	7.87
Object detection and segmentation	2	4	0	6	0.5	15.88
Prognosis detection	0	1	0	1	3.0	0.0
Surgical task analysis	1	1	0	2	3.7	11.3
Medical image analysis	1	4	0	5	2.0	1.7

*IQR,* Interquartile range.

**Table E2:** Imaging (domain × metric)

Domain	Metric	n	T>NT	Tie	T<NT	Median Δ (percentage points)	IQR Δ (percentage points)
Education: Medical diagnosis	Precision	1	0	0	1	−0.52	0.0
Education: Medical diagnosis	Recall	1	1	0	0	0.38	0.0
Education: Surgery time prediction	Diagnostic accuracy	3	3	0	0	5.57	9.16
Education: Surgery time prediction	Precision	1	1	0	0	0.97	0.0
Object detection and segmentation	Diagnostic accuracy	2	1	0	1	−1.95	2.05
Object detection and segmentation	F1 score	1	1	0	0	17.3	0.0
Object detection and segmentation	Precision	2	1	0	1	−1.35	2.25
Object detection and segmentation	Recall	1	1	0	0	18.3	0.0
Prognosis detection	Diagnostic accuracy	1	1	0	0	3.0	0.0
Surgical task analysis	Diagnostic accuracy	2	1	0	1	3.7	11.3
Medical image analysis	Diagnostic accuracy	2	1	0	1	0.7	1.3
Medical image analysis	F1 score	1	1	0	0	2.1	0.0
Medical image analysis	Precision	2	2	0	0	1.4	1.0

*IQR,* Interquartile range; *F1 score,* Harmonic mean of precision and recall.

**Table E3:** Workflow (domain × metric)

Domain	Metric	n	T>NT	Tie	T<NT	Median Δ (percentage points)	IQR Δ (percentage points)
Education: Clinical documentation	Diagnostic accuracy	1	1	0	0	27.4	0.0
Education: Clinical documentation	F1 score	2	2	0	0	3.3	1.8
Education: Medical diagnosis	Precision	1	0	0	1	−0.52	0.0
Education: Surgical automation	Diagnostic accuracy	5	5	0	0	3.9	4.39
Education: Surgical Automation	F1 score	1	1	0	0	1.28	0.0
Education: Surgical automation	Precision	3	3	0	0	3.4	3.42
Object detection and segmentation	Diagnostic accuracy	2	1	0	1	0.27	4.27
Object detection and segmentation	F1 score	1	1	0	0	17.3	0.0
Object detection and segmentation	Precision	2	1	0	1	−1.35	2.25
Prognosis detection	Diagnostic accuracy	1	1	0	0	3.0	0.0
Surgical task analysis	Diagnostic accuracy	3	1	0	2	−0.13	4.91
Surgical task analysis	Precision	2	2	0	0	1.26	0.02
Medical image analysis	Diagnostic accuracy	5	4	0	1	0.9	1.4
Medical image analysis	F1 score	2	2	0	0	2.6	0.5
Medical image analysis	Precision	3	3	0	0	2.4	1.95

*IQR,* Interquartile range; *F1 score,* Harmonic mean of precision and recall.
